# Risk Perception Related to COVID-19 and Future Affective Responses Among Healthcare Workers in Switzerland: A Mixed-Methods Longitudinal Study

**DOI:** 10.3389/ijph.2022.1604517

**Published:** 2022-09-02

**Authors:** Véronique S. Grazioli, Konstantinos Tzartzas, Jérémie Blaser, Madison Graells, Elodie Schmutz, Isabelle Petitgenet, Bernard Favrat, Javier Sanchis Zozaya, Ioannis Kokkinakis, Regis Marion-Veyron, Patrick Bodenmann

**Affiliations:** Center for Primary Care and Public Health, University Center of General Medicine and Public Health, Lausanne, Switzerland

**Keywords:** anxiety, burnout, healthcare workers, depression, risk perceptions related to COVID-19, secondary traumatic stress

## Abstract

**Objectives:** Whereas early findings suggest that risk perceptions related to COVID-19 affect psychological well-being in healthcare workers (HCWs), the temporal associations between these variables need to be clarified and HCWs lived experience further explored. This study proposes a mixed evaluation of COVID-19-related risk perception and affective responses among HCWs.

**Methods:** A longitudinal mixed-method study was conducted. HCWs (*N* = 138) completed measures of COVID-19 risk perceptions, depression, anxiety, burnout and secondary traumatic stress (STS) at baseline and 6 months later. A subsample (*n* = 20) participated in semi-structured interviews exploring both risk perceptions and affective responses.

**Results:** Main quantitative findings showed positive associations between worry to contaminate others and depression (*IRR* = 1.04, *p* < 0.05), anxiety (*IRR* = 1.03, *p* < 0.01), STS (*b* = 0.3, *p* < 0.05), and perceptions of lacking protection (*IRR* = 1.04, *p* < 0.05) with anxiety scores. Four themes emerged from the thematic content analysis: 1) life was turned upside down; 2) skills were put in quarantine; 3) dealing with patient discomfort; 4) balance to be found between protection and restrictions.

**Conclusion:** These findings emphasize the importance to develop tailored interventions, such as group discussion sessions, to optimize risk perception and help manage uncertainty.

## Introduction

The outbreak of coronavirus disease 2019 has led to unprecedented challenges around the world, reaching 220 countries and territories, with up to 517,648,631 confirmed cases and 6,261,708 deaths worldwide (situation report updated to 10th May 2022) [1]. This unprecedented situation placed healthcare workers (HCWs) at high risk to experience affective responses [2, 3]. In fact, previous findings indicate that HCWs commonly experienced depression, anxiety, burnout (i.e., response to chronic stress at workplace, including emotional exhaustion, depersonalization and capacities reduction) [4] and secondary traumatic stress symptoms (i.e., STS; compassion fatigue and stress resulting from helping others) [5], during the COVID-19 outbreak [6–12].

Interestingly, not all HCWs experience affective responses at the same level, frontline HCWs (i.e., working directly with suspected or confirmed cases), females and nurses being at higher risk to report affective responses [6–8, 13]. In addition, the way in which HCWs’ psychological well-being is affected varies according to the phase of the pandemic. In the first phase, the fears, the uncertainty and the increased workload predominated [14, 15], whereas afterwards, compassion fatigue and stress resulting from the new way of helping others emerged [16].

Another factor associated with affective response levels is risk perception (i.e., reflexive process by which individuals identify and give meaning to an event as a threat) [17]. According to two studies conducted in Europe during COVID-19 outbreak, 59% HCWs worried about being infected and contaminate patients and relatives [18, 19]. Importantly, cross-sectional studies consistently revealed positive associations between risk perception related to COVID-19 and affective responses among HCWs, such as anxiety, PTSD, depression and stress symptoms [2, 3]. Other previous cross-sectional research examined these associations among HCWs who were not frontline providers and found similar findings. A study conducted in Finland showed that healthcare and social workers who perceived higher risk of COVID-19 infection in their workplace reported stress symptoms more often than those who reported lower risk perception [20]. Similar positive associations were found between risk perception and depression, anxiety and stress symptoms among HCWs (health centres of Qatar Red Crescent Society) [21], dentists and HCWs from hospitals in Italy [13, 22], in China and in Hong Kong [23].

These studies provide initial evidence of a cross-sectional association between risk perception related to COVID-19 and affective responses among HCWs. However, longitudinal research is needed to draw conclusions about the temporal association between these events. Furthermore, to the best of the authors’ knowledge, only three qualitative studies conducted in Oman, in Australia and in the US among HCWs explored these dimensions [24, 25]. Findings documented that HCWs were afraid of being exposed to COVID-19 and contaminate others, which was exacerbated by the shortage of infection prevention measures. Further in-depth qualitative research exploring HCWs’ lived experience is needed to gain a finer understanding of their risk perception and affective responses, provide insight into the interpretation of quantitative results and ultimately identify what well-tailored support could temper such reactions.

In response, this longitudinal mixed-method study aimed to 1) evaluate the associations between risk perception related to COVID-19 and depression, anxiety, burnout and STS symptoms 6 months later, 2) explore risk perception and affective responses qualitatively among HCWs in Switzerland. Based on the cross-sectional evidence, we hypothesized that higher risk perception levels at baseline would be significantly associated with higher affective responses 6 months later.

## Methods

### Participants

Participants were HCWs working at the Center for Primary Care and Public Health in the French part of Switzerland (Unisanté). Inclusion criteria were being part of the medical (i.e., primary care physicians) or non-medical HCWs (e.g., nurses, pharmacists, psychologists); working at Unisanté during the COVID-19 first wave (i.e., February-June 2020). In total, 339 staff were eligible and invited to participate (i.e., 69.5% female). Of those, 178 participants (52.3%) completed the baseline assessment and 138 completed the 6-month follow-up assessment (77.5%). A 6-month follow-up period was deemed reasonable given the rapidity with which knowledge regarding COVID-19 transmission arose.

### Procedures

#### Recruitment and Quantitative Assessment

Eligible staff were invited by email to participate in the study. Interested HCWs logged in to an electronic case report form to proceed to the informed and written consent. Then, participants received an email with a unique identifier linking to the baseline questionnaire. Six months later, participants received an email inviting them to complete the follow-up assessment.

#### Qualitative Assessment

Three-month post-baseline, participants were invited to take part to a semi-structured interview. To obtain maximum variation sampling, three professional groups were selected (primary care physicians, nurses and pharmacists). The research staff randomly selected participants from each professional status until data saturation was achieved. Interviews were conducted by three senior researchers with experience in qualitative assessment and mental health. After the first 20 interviews, the investigators combined their experiences and noticed the repetition of similar comments, agreeing that data saturation had been reached. Interviews were conducted face-to-face or by phone, and lasted between 32 and 77 min. Sessions were audiotaped and fully transcribed.

#### Ethical Approval

All procedures were conducted in accordance with the ethical standards of the Human Research Ethics Committee of Canton de Vaud (CER-VD, 2020-00967).

### Quantitative Measures

#### Demographic Variables

Age, gender and professional activity (work) were assessed at baseline. Two items evaluated psychological status prior to COVID-19 pandemic (ongoing psychotherapy, psychotropic drug prescription), recoded as 1 (answering “yes” to at least one question) and 0 (answering “no” to both questions).

#### Risk Perception Related to COVID-19

Risk perception over the past 2 months was assessed with a measure developed in the context of Ebola that was adapted to COVID-19 [26]. Participants were asked to indicate their perceptions of exposure to COVID-19 and concerns for infection, transmission and protective measure effectiveness. The items were subject to a principal component analysis (PCA). Suitability of data for PCA was supported by correlation matrix inspection revealing coefficients of 0.3 and above, value of Kasier-Meyer-Olkin (0.72) and statistical significance of Bartlett’s Test of Sphericity (*p* < 0.001). PCA revealed 3 components with eigenvalues exceeding one, explaining 27.14%, 15.29%, 12.44%, of the variance: 1) risk perceptions to contract COVID-19 (Risk; *α* = 0.72, men inter-item = 0.41); 2) perceptions of lacking protection (Unprotect; *α* = 0.56, mean inter item = 0.31); and 3) worry to contract COVID-19 and consequences (Worry; *α* = 0.61, mean inter item = 0.28).

#### Depression and Anxiety Symptoms

Depression and symptoms over the past 2 weeks were assessed with the Quick Inventory of Depressive Symptomatology Self-Report (QIDS-SR) [27] and the Generalized Anxiety Disorder 7-item (GAD-7) [28]. Internal consistency was adequate (*α* = 0.75 and 0.88).

#### Job Burnout and STS

Job burnout and STS symptoms were assessed with two subscales of the Professional Quality of Life Scale (PrQoL) [29]. Job burnout (*α* = 0.70) and STS (*α* = 0.78) showed adequate internal consistencies.

### Qualitative Measure

Semi-structured interviews were guided by a grid aiming to explore HCWs’ experience, emotional responses, risk perception, encountered difficulties and strategies used to address difficulties (see Supplementary Appendix S1).

### Data Preparation and Analysis Plan

#### Quantitative Analysis

Depression (*S* = 1.8, *K* = 1.13) and anxiety scores (*S* = 0.9, *K* = 1.14) showed distributions approximating a negative binomial and a Poisson distribution, respectively. Thus, negative binomial and Poisson regressions were used to test the associations of risk perception with depression and anxiety scores. Linear regressions were selected for burnout (*S* = −0.04, *K* = 0.06) and STS (*S* = 0.67, *K* = 0.98) scores that followed normal distributions.

Risk perception predictors (i.e., unprotect, worry and risk) were tested in the same model, resulting in four finale models (i.e., depression, anxiety, burnout and STS score models). All participants had at least indirect contacts with patients with COVID-19, whereas most had direct contacts as well. Given the known associations between being frontline workers, risk perception and affective responses [13, 30] we first conducted analyses with the whole sample. Then, we repeated them excluding participants reporting indirect contacts only. All models were adjusted for age, gender, work and psychological status. Analyses were conducted with STATA 16. The significance level was set at *p* = 0.05.

#### Qualitative Analysis

Inductive thematic content analysis was conducted. Initially, KT with MG (psychologist) independently coded the same “raw data.” Emerging codes were compared to establish the “extent of possible overlap,” different “sets” of codes having been combined or reorganized. Once the new codes were established, they were confronted by a third researcher, VG (psychologist). New codes were incorporated to the codebook created during consensus meetings [31]. We used the codebook to independently double-code 20% of data until adequate intercoder consistency was established and the remaining interviews were coded independently [32]. Afterwards, KT explored overarching themes, which were presented to the research team to confirm interpretation. The teams’ feedback was integrated to analysis [33]. Data management and analysis was facilitated by the software ATLAS.ti.

## Results

### Quantitative Results: Study Sample Description and Descriptive Statistics

The sample (*N* = 138) mean age was 40.83 (*SD* = 11.33) and 75% self-identified as female. Almost half participants were physician, whereas 25% were nurses and 28% others (e.g., pharmacists). The prevalence of females and professions are close to those in the home institution. Table 1 presents descriptive statistics for key variables.

**TABLE 1:** Descriptive Statistics and Significance by Assessment Time (Risk perception related to COVID-19 and future affective responses among healthcare workers in Switzerland: A mixed-methods longitudinal study; Switzerland, 2022).

	Baseline		6-month follow-up		
	Mdn	IQR	Mdn	IQR	*Z*
Depression score	4	5	4.5	6	1.76
Anxiety score	3	5	3.5	7	3.11**
	M	SD	M	SD	*t*
Burnout	21.37	5.18	22.06	5.76	−1.73***
STS	18.4	5.19	18.36	5.58	0.11
Unprotect	8	2.73	9.28	2.66	−5.29***
Worry	16.81	3.98	16.98	3.59	−0.54
Risk	16.14	4.72	16.24	4.39	−0.26

*p< 0.05; **p< 0.01; ***p< 0.001.

### Attrition Analyses

Attrition analyses indicated that non-completers (*M* = 37.13, *SD* = 10.75) at 6 months were significantly younger than completers (*M* = 42.01, SD = 11.54; *p* = 0.02). Therefore, analyses were adjusted for age. Remaining attrition analyses on psychological status, work, risk perceptions and affective response scores found no significant differences.

### Regression Models Predicting Depression, Anxiety, Burnout and STS Scores in Participants Reporting Indirect and Direct Contacts With Infected Patients

Table 2 displays results from the unadjusted and adjusted models.

**TABLE 2:** Regression Models Predicting Depression, Anxiety, Burnout and Secondary Traumatic Stress Scores in the Whole Sample (*N* = 138; Risk perception related to COVID-19 and future affective responses among healthcare workers in Switzerland: A mixed-methods longitudinal study; Switzerland, 2022).

	Depression score^a^			Anxiety score^b^		
	IRR (SE)	*Z*	95% CI	IRR (SE)	*Z*	95% CI
Unadjusted models						
Unprotect	1.05* (0.03)	2.06	1.01, 1.11	1.05*** (0.01)	3.5	1.02, 1.08
Worry	1.04* (0.02)	2.30	1.01, 1.08	1.03** (0.01)	3.21	1.01, 1.05
Risk	1.01 (0.02)	0.56	0.98, 1.04	1.04 (0.01)	1.24	0.99, 1.03
Adjusted models^c^						
Unprotect	1.05 (0.03)	1.82	0.99, 1.11	1.04* (0.02)	2.51	1.01, 1.07
Worry	1.04* (0.02)	2.15	1.01, 1.08	1.03** (0.01)	2.83	1.01, 1.05
Risk	1.02 (0.01)	0.86	0.98, 1.05	1.01 (0.01)	1.23	0.99, 1.03
	Burnout score^b^			Secondary traumatic stress score^b^		
	*B* (SE)	*T*	95% CI	*B* (SE)	*t*	95% CI
Unadjusted models						
Unprotect	0.44* (0.18)	2.41	0.08, 0.79	0.23 (0.18)	1.31	−0.12, 0.58
Worry	0.25* (0.13)	2.01	0.01, 0.5	0.31* (0.12)	2.47	0.06, 0.55
Risk	−0.15 (0.11)	−1.39	−0.37, 0.07	0.02 (0.11)	0.16	−0.20, 0.23
Adjusted models^c^						
Unprotect	0.37 (0.20)	1.87	−0.02, 0.76	0.19 (0.18)	1.03	−0.17, 0.55
Worry	0.25 (0.13)	1.9	−0.01, 0.051	0.3* (0.2)	2.51	0.06, 0.54
Risk	−0.11 (0.12)	−0.94	−0.6, 0.13	0.1 (0.11)	0.88	−0.13, 0.33

a Negative binomial regression.

b Poisson regression.

c Models were adjusted for age, gender, work and psychological status.

*p< 0.05; **p< 0.01; ***p< 0.001.

#### Depression Score

The likelihood ratio for the adjusted negative binomial model was *X* ^2^ [8] = 16.49, *p* < 0.05. Worry was related to higher depression score over time (*IRR* = 1.04, *p* < 0.05), whereas the associations of unprotect (*IRR* = 1.05, *p* > 0.05) and risk (*IRR* = 1.02, *p* > 0.05) with depression score were not significant.

#### Anxiety Score

The likelihood ratio for the adjusted model 1 was *X* ^2^ [8] = 44.81, *p* < 0.001. Unprotect (*IRR* = 1.04, *p* < 0.05) and worry (*IRR* = 1.03, *p* < 0.01) were associated with higher anxiety scores over time. The association between risk (*IRR* = 1.01, *p* > 0.05) and anxiety score were not significant.

#### Burnout Score

The adjusted model was not significant (F [8, 126] = 1.3, *p* = 0.3). Likewise, unprotect (*b* = 0.37, *p* > 05), worry (*b* = 0.25, *p* > 0.05) and risk (*b* = -0.11, *p* > 0.05) were not significantly associated with burnout scores.

#### STS Score

The adjusted model was significant (F [8, 125] = 3.26, *p* = 0.002). The associations of unprotect (*b* = 0.19, *p* > 0.05) and risk (*b* = 0.1, *p* > 0.05) with STS scores were not significant. There was however a positive association between worry (*b* = 0.3, *p* < 0.05) and subsequent STS scores.

### Regression Models Predicting Depression, Anxiety, Burnout and STS Scores Excluding Participants Reporting No Direct Contacts With Infected Patients

We present below results from the adjusted models including frontline HCW (*n* = 108; see Table 3).

**TABLE 3:** Regression Models Predicting Depression, Anxiety, Burnout and Secondary Traumatic Stress Scores in Participants Reporting Direct Contacts with Infected Patients (*n* = 104; Risk perception related to COVID-19 and future affective responses among healthcare workers in Switzerland: A mixed-methods longitudinal study; Switzerland, 2022).

	Depression score^a^			Anxiety score^b^		
	IRR (SE)	*Z*	95% CI	IRR (SE)	Z	95% CI
Unadjusted models						
Unprotect	1.06* (0.03)	2.32	1.01, 1.13	1.06*** (0.01)	4.08	1.03, 1.09
Worry	1.05* (0.02)	2.49	1.01, 1.09	1.05*** (0.01)	4.43	1.03, 1.07
Risk	0.98 (0.02)	−0.72	0.94, 1.03	0.96** (0.01)	−3.31	0.94, 0.98
Adjusted models^c^						
Unprotect	1.06* (0.03)	1.97	1.00, 1.12	1.06*** (0.02)	3.53	1.03, 1.1
Worry	1.04* (0.02)	2.23	1.01, 1.08	1.04*** (0.01)	3.91	1.02, 1.06
Risk	0.99 (0.02)	−0.67	0.94, 1.03	0.96** (0.01)	−3.19	0.94, 0.98
	Burnout score^b^			Secondary traumatic stress score^b^		
	*B* (SE)	*t*	95% CI	*B*(SE)	*t*	95% CI
Unadjusted models						
Unprotect	0.53** (0.2)	2.69	0.14, 0.93	0.33 (0.2)	1.64	−0.07, 0.73
Worry	0.34* (0.13)	2.55	0.08, 0.6	0.4**(0.14)	2.71	0.1, 0.64
Risk	−0.49** (0.15)	−3.34	−0.79, −0.2	−0.29 (0.15)	−1.95	−0.59, 0.004
Adjusted models^c^						
Unprotect	0.54* (0.22)	2.45	0.1, 0.97	0.33 (0.21)	1.55	−0.09, 0.74
Worry	0.31* (0.14)	2.26	0.04, 0.6	0.4** (0.13)	2.88	0.12, 0.64
Risk	−0.48** (0.16)	−2.94	−0.8, −0.15	−0.3 (0.15)	−1.54	−0.55, 0.07

a Negative binomial regression.

b Poisson regression.

c Models were adjusted for age, gender, work and psychological status.

*p< 0.05; **p< 0.01; ***p< 0.001.

#### Depression Score

The likelihood ratio for the adjusted model was *X* ^2^ [8] = 16.02, *p* < 0.05. Unprotect (*IRR* = 1.06, *p* < 0.05) and worry (*IRR* = 1.04, *p* < 0.05) at baseline were related to higher depression scores at 6 months, whereas the association between risk (*IRR* = 0.99, *p* > 0.05) and depression scores was not significant.

#### Anxiety Score

The likelihood ratio for the adjusted model was *X* ^2^ [8] = 52.6, *p* < 0.001. Unprotect (*IRR* = 1.06, *p* < 0.001) and worry (*IRR* = 1.04, *p* < 0.001) were associated with higher anxiety scores over time, whereas risk (*IRR* = 0.96, *p* < 0.01) was related to subsequent decreased anxiety scores.

#### Burnout Score

The adjusted model was significant (F [8, 96] = 2.9, *p* = 0.006). Unprotect (*b* = 0.54, *p* < 0.05) and worry (*b* = 0.31, *p* < 0.05) were related to higher scores, whereas risk (*b* = -0.48, *p* < 0.01) was associated with decreased burnout scores.

#### Secondary Traumatic Stress

The adjusted model was significant (F [8, 96] = 3.44, *p* = 0.001). The associations of unprotect (*b* = 0.33, *p* > 0.05) and risk (*b* = −0.3, *p* > 0.05) with STS scores were not significant. There was however a positive association between worry (*b* = 0.4, *p* < 0.01) and subsequent STS scores.

### Qualitative Results: Study Sample Description and Emerging Themes

Twenty participants carried out a qualitative interview (85% female; mean age = 40.55; 35% of physician, 35% nurses, 30% pharmacists). The higher female prevalence is consistent with the higher non-physician healthcare workers in this subsample (i.e., in the home institution: 53.8% female among physicians; 88% female among other healthcare workers). Four main themes emerged regarding HCWs’ lived experience and risk perception when facing COVID-19 pandemic. The thematic framework arising through thematic analysis is displayed in Table 4.

**TABLE 4:** Thematic Framework Highlighting the Major Themes and Subthemes (Risk perception related to COVID-19 and future affective responses among healthcare workers in Switzerland: A mixed-methods longitudinal study; Switzerland, 2022).

Major themes and subthemes summarizing HCWs’ lived experiences regarding risk perception related to COVID-19 and affective responses during the first wave
Theme 1: Life was turned upside down
Sub-themes
• Major changes in daily activities
• Ambivalent feelings towards their profession
• In a never-ending crisis process
• A sacrificial stance
Theme 2: Skills were put in quarantine
Sub-themes
• Confrontation with various limits
• A sense of lack of choice
Theme 3: Dealing with patient discomfort
Sub-themes
• Facing patients’ panic
• Healthcare workers’ anxiety
Theme 4: Balance to be found between protection and restrictions
Sub-themes
• Reassurance regarding protective measures
• Frustration towards protective measures
• Fear of infecting others
• A need for direct contacts

### Life Was Turned Upside Down

#### Major Changes in Daily Activities

A strong feeling that daily activities were entirely modified during the first weeks of the pandemic was pointed out by the participants. They commonly said that “everything was different,” having a feeling of practicing “a different sort of medicine.” In their private life, a new complexity emerged, which led to strong feelings of uncertainty. They described that they “had to reorganize everything”: “We coped as well as we could; … We had to organize ourselves completely differently… I was teleworking in the morning, I slept a little more in the morning, I had lunch in front of the computer…”

#### Ambivalent Feelings Towards Their Profession

Being a HCW during the pandemic provoked a feeling of valorization as well as a societal pressure. This was reflected in the ambiguous attitude of participants’ relatives: “We were asked a lot of questions; and then when we didn’t know, they started to transform … in the beginning, I saw how scared my parents were saying: “But did you wash your hands well when you arrived?” Certain times, HCWs had the impression of being “seen as pestiferous.” An overall impression of having been “pushed out of the comfort zone” was noted.

#### In a Never-Ending Crisis Process

In addition, the multitude of constantly changing information and guidelines made it hard for them to keep-up, provoking a feeling of being “stuck in a crisis process” that never ended. They mentioned experiencing a continuous presence of a “sword of Damocles,” anticipating being overwhelmed by the situation: “We were seeing the images of China with the caregivers who were crying in the corners and having their anxiety attacks in the room… I was like, “Ouch!” I … I don’t really want to go in there, I was terrified…”

#### A Sacrificial Stance

Facing this situation, participants described having deployed multiple resources a “savior” posture emerging, with a sacrifice of their “basic needs” to bring their help during an exceptional situation: “I was kind of someone who came in...not to save the situation but, almost!”

### Skills Were Put in Quarantine

#### Confrontation With Various Limits

Participants mentioned that they faced various shortcomings, facing their own professional limits (i.e., lack of knowledge, preparation and experience to deal with the situation) and not being able to deploy their usual skills. Feelings of powerlessness and being “out-of-step” were noted, hoping that they “do the right thing” (e.g., “I think there was a lot of anxiety and fear at the beginning, regarding the unknown”). At the same time, participants underlined that their superiors and the whole institution could not anticipate this condition. Lack of equipment for protection or teleworking, lack of time to get organized, lack of drugs, clear information and communication adjusted to their needs were experienced, causing feelings of insecurity and vagueness: “The hierarchy, …, they know that we are going to miss masks, we are going to miss gowns, we are going to miss respirators… and they try to find solutions…, they do what they can.”

#### A Sense of Lack of Choice

A destabilizing feeling of “not having a choice” was evoked, HCWs describing having to prioritize COVID-19 activities, to make inquiries or to work overtime*.* Most of all, they were afraid of being overwhelmed:

I could really see myself running out of … of caregivers, out of equipment. And this kind of approach was stressing me out: so, at work, I didn’t want to experience this, to live through this… this increasing number of patients…

### Dealing With Patient Discomfort

#### Facing Patients’ Panic

They described that their patients were concerned about a possible contamination risk, but also about the evolution of the pandemic and more globally about their socio-professional situation, feeling by moments “ultra-panicked.” HCWs had to provide both medical evaluation and reassurance, doing “a lot of psychiatric support”: “When there was a lot of uncertainty and people were psychologically distressed, it was a heavy workload; with regard mostly to psychiatric cases … actually, for mental health.”

#### Healthcare Workers’ Anxiety

Participants themselves faced multiple professional difficulties that impinged on their capacity to offer usual care, circumstances that made their task even more complex: “Having to respond to their anxiety while I was myself anxious, and then constantly in this state of uncertainty, was really difficult…” Furthermore, the fact that most interviews took place at distance, but also the cancellation of the organized follow-ups, were some of the factors that put the therapeutic relationship in difficulty.

### Balance to Be Found Between Protection and Restrictions

#### Reassurance Regarding Protective Measures

Once the equipment was available and the protective measures applied, participants reported feeling well protected. The “barrier gestures” became part of their routine, which reduced their sense of fear and anxiety in their work environment.

#### Frustration Towards Protective Measures

At the same time, frustration feelings emerged, especially regarding the lack of work breaks, group discussions and direct contact. In private life they described that it was difficult to find a balance between taking risks and protecting themselves, provoking a continuous stress.

#### Fear of Infecting Others

On the one hand, the fear of being infected, but above all, of infecting relatives or people at risk, was highlighted. They were also thinking about the risk of overloading the health care system or their own team, if they were contaminated. A strong need to avoid feeling guilty was expressed: “I was more worried about transmitting it to other people that they would get sick because of me…”

#### A Need for Direct Contacts

On the other hand, they described that restrictions were hard to bear. The need to be in contact with relatives and colleagues arose, enforced relational distance and a lack of social life being not easily tolerated. Therefore, the experience of a tense atmosphere was voiced, participants having the feeling that it was impossible to always follow the prescribed measures: “We must stop this psychosis… we didn’t go to restaurants, we didn’t go out, we didn’t see anyone…” They described that they had to continuously navigate between feelings of frustration regarding restrictions and fear of contamination relieved by those same restrictions.

## Discussion

To our knowledge, this is the first mixed-method longitudinal study investigating the association between risk perception related to COVID-19 and affective responses in HCWs. Quantitative findings indicated that higher levels of worry to contaminate others were related to higher depression, anxiety and STS scores over time, whereas the same association was found for burnout score for frontline participants only. Positive associations between perceptions of lacking protection and future anxiety score were revealed, whereas the same link was found for depression and burnout scores among frontline participants only. Finally, unexpectedly, higher levels of risk perception to contract COVID-19 was associated with decreased levels of anxiety and burnout over time among frontline participants.

Concerning qualitative findings, at first, participants indicated that both their professional and private “life was turned upside down.” They faced continuous and rapid changes, finding themselves in a never-ending crisis process, which provoked a sacrificial stance, while facing shortcomings and feeling their usual professional “skills were put in quarantine.” “Dealing with patient discomfort” was also challenging, reinforced by participants’ anxiety, professional difficulties and lack of direct contact with their patients. Once protective measures were applied and a certain sense of security established, frustration feelings emerged and a new “balance had to be found between protection and restrictions.”

Consistent with earlier research, worrying about being infected and contaminate others were central issues for HCWs [18, 19, 25]. Our results add to the literature by showing that higher levels of worry were associated with subsequent higher depression, anxiety and STS scores [34]. These associations arose with HCWs’ feeling of not having a choice but to prioritize COVID-19 activities, while having little knowledge about the impact of a potential infection and the feeling that their relatives had an ambiguous attitude towards them [35]. These challenges may have induced internal conflicts between HCWs’ devotion to their work [36] and an urge to protect loved ones [34], potentially resulting in negative affective reactions over time [16].

These conflicts could be amplified among frontline workers, known to have higher rates of affective responses than non-frontline HCWs [6–8, 13]. Consistently, we found that higher levels of worry and unprotect perceptions were associated with higher burnout scores over time among frontline HCWs. Participants stated that they were in constant crisis process, working overtime, with multiple changes in their work context, a situation described as insecure and overwhelming [24, 36, 37]. They also evoked a tendency to adopt a “savior” posture, sacrificing their “basic needs,” which could undermine their health at work.

Contrary to past findings showing positive associations of perception to contract COVID-19 with anxiety and burnout symptoms [2, 3, 9, 20, 21, 23], our findings indicated that risk perception to contract COVID-19 was associated with decreased levels of anxiety and burnout in frontline HCWs over time. Our findings are yet congruent with recent longitudinal research focusing on affective responses that revealed positive evolutions over time [16]. It may be that frontline HCWs faced uncertainty in the beginning of the outbreak due to a lack of knowledge and shortcomings in protective equipment, all of which improved after 6 months. We can hypothesize that by facing this extreme situation, they acquired knowledge and experience, progressively adjusted their risk perception, adopting the necessary protection, and ultimately felt safer [36]. It is possible that such experience, together with institutional organizations, made them feel relieved from work-related distress, which emerged from our qualitative inquiries and was also evoked in existing literature [7]. We could also imagine that frontline HCWs could more easily handle the ambivalence between risk-taking and self-protection [38] once they stepped out of acute crises [35]. An adjusted risk perception could therefore be a key element to consider, helping finding a balance between the need to establish a secure environment and feelings of frustration about restrictions.

Our study sheds more light to multiple associations between risk perception related to COVID-19 and affective responses among HCWs. Stakeholders and mental health professionals are called upon to provide HCWs with interventions to deal with their emerging affective reactions, considering their worries and internal ambivalences. As the pandemic continues, tailored individual and structural interventions should be offered [36], such as group or individual discussion sessions, promoting mutual support among HCWs and counteracting social isolation [37].

The main limitation of this study relates to the specificity of the local healthcare system, limiting the generalizability of our findings to healthcare systems stemming from other countries, although the Swiss healthcare system was severely challenged as it was the case in most healthcare systems worldwide [34]. Sampling bias may also present, as HCWs who responded to the survey may differ from those who did not, although we ensured that all different professions within the institution were represented. Finally, the fact that 75% of the participants identified themselves as female could represent a bias, even though this prevalence was close to the one in the home institution (i.e., 69%) and consistent to those described in previous studies [8].

This study increases our knowledge of the associations between HCWs’ risk perception and affective responses over time. Combined and interpreted with our qualitative inquiries, results emphasize the importance to develop individual and structural support interventions to optimize risk perception and minimize affective responses development over time among HCWs.

## References

[1] WHO. Coronavirus Disease (COVID-19) Situation Report—171 (2020). Available from https://covid19.who.int/ (Accessed May 10, 2022).

[2] Yin Q Chen A Song X Deng G Dong W. Risk Perception and PTSD Symptoms of Medical Staff Combating against COVID-19: A PLS Structural Equation Model. Front Psychiatry (2021) 12:607612. 10.3389/fpsyt.2021.607612 33658951 PMC7917132

[3] Yildirim M Arslan G Ozaslan A. Perceived Risk and Mental Health Problems Among Healthcare Professionals during COVID-19 Pandemic: Exploring the Mediating Effects of Resilience and Coronavirus Fear. Int J Ment Health Addict (2020) 20:1035–45. 10.1007/s11469-020-00424-8 33223977 PMC7668285

[4] Maslach C Jackson SE. The Measurement of Experienced Burnout. J Organ Behav (1981) 2:99–113. 10.1002/job.4030020205

[5] Figley CR. Catastrophes: An Overview of Family Reactions. In: M Brunner, editor. Stress and the Family. New York, USA (1983). p. 3–20.

[6] Pappa S Ntella V Giannakas T Giannakoulis VG Papoutsi E Katsaounou P. Prevalence of Depression, Anxiety, and Insomnia Among Healthcare Workers during the COVID-19 Pandemic: A Systematic Review and Meta-Analysis. Brain Behav Immun (2020) 88:901–7. 10.1016/j.bbi.2020.05.026 32437915 PMC7206431

[7] Shreffler J Petrey J Huecker M. The Impact of COVID-19 on Healthcare Worker Wellness: A Scoping Review. West J Emerg Med (2020) 21(5):1059–66. 10.5811/westjem.2020.7.48684 32970555 PMC7514392

[8] Vizheh M Qorbani M Arzaghi SM Muhidin S Javanmard Z Esmaeili M. The Mental Health of Healthcare Workers in the COVID-19 Pandemic: A Systematic Review. J Diabetes Metab Disord (2020) 19:1967–78. 10.1007/s40200-020-00643-9 33134211 PMC7586202

[9] Lasalvia A Amaddeo F Porru S Carta A Tardivo S Bovo C Levels of Burn-Out Among Healthcare Workers during the COVID-19 Pandemic and Their Associated Factors: a Cross-Sectional Study in a Tertiary Hospital of a Highly Burdened Area of north-east Italy. BMJ Open (2021) 11(1):e045127. 10.1136/bmjopen-2020-045127 PMC7813385 33455940

[10] Orru G Marzetti F Conversano C Vagheggini G Miccoli M Ciacchini R Secondary Traumatic Stress and Burnout in Healthcare Workers during COVID-19 Outbreak. Int J Environ Res Public Health (2021) 18(1):E337. 10.3390/ijerph18010337 33466346 PMC7794988

[11] Hu D Kong Y Li W Han Q Zhang X Zhu LX Frontline Nurses' Burnout, Anxiety, Depression, and Fear Statuses and Their Associated Factors during the COVID-19 Outbreak in Wuhan, China: A Large-Scale Cross-Sectional Study. EClinicalMedicine (2020) 24:100424. 10.1016/j.eclinm.2020.100424 32766539 PMC7320259

[12] Magnavita N Chirico F Garbarino S Bragazzi NL Santacroce E Zaffina S Association of Occupational Distress and Low Sleep Quality with Syncope, Presyncope, and Falls in Workers. Int J Environ Res Public Health (2021) 18(8):12283. 10.3390/ijerph182312283 34886008 PMC8657064

[13] Gorini A Fiabane E Sommaruga M Barbieri S Sottotetti F La Rovere MT Mental Health and Risk Perception Among Italian Healthcare Workers during the Second Month of the Covid-19 Pandemic. Arch Psychiatr Nurs (2020) 34(6):537–44. 10.1016/j.apnu.2020.10.007 33280678 PMC7577253

[14] Magnavita N Tripepi G Di Prinzio RR. Symptoms in Health Care Workers during the COVID-19 Epidemic. A Cross-Sectional Survey. Int J Environ Res Public Health (2020) 17(14):E5218. 10.3390/ijerph17145218 32698320 PMC7400440

[15] Magnavita N Soave PM Ricciardi W Antonelli M. Occupational Stress and Mental Health Among Anesthetists during the COVID-19 Pandemic. Int J Environ Res Public Health (2020) 17(21):E8245. 10.3390/ijerph17218245 33171618 PMC7664621

[16] Magnavita N Soave PM Antonelli M. A One-Year Prospective Study of Work-Related Mental Health in the Intensivists of a COVID-19 Hub Hospital. Int J Environ Res Public Health (2021) 18(18):9888. 10.3390/ijerph18189888 34574811 PMC8466101

[17] Vohra S. Risk Perception and Communication 2003.

[18] Puci MV Nosari G Loi F Puci GV Montomoli C Ferraro OE. Risk Perception and Worries Among Health Care Workers in the COVID-19 Pandemic: Findings from an Italian Survey. Healthcare (Basel) (2020) 8(4):E535. 10.3390/healthcare8040535 33287260 PMC7761765

[19] Riguzzi M Gashi S. Lessons from the First Wave of COVID-19: Work-Related Consequences, Clinical Knowledge, Emotional Distress, and Safety-Conscious Behavior in Healthcare Workers in Switzerland. Front Psychol (2021) 12:628033. 10.3389/fpsyg.2021.628033 33633652 PMC7899962

[20] Finell E Vainio A. The Combined Effect of Perceived COVID-19 Infection Risk at Work and Identification with Work Community with Psychosocial Wellbeing Among Finnish Social Sector and Health Care Workers. Int J Environ Res Public Health (2020) 17(20):E7623. 10.3390/ijerph17207623 33086738 PMC7589430

[21] Abed Alah M Ali K Abdeen S Al-Jayyousi G Kasem H Poolakundan F The Psychological Impact of COVID-19 on Health Care Workers Working in a Unique Environment under the Umbrella of Qatar Red Crescent Society. Heliyon (2021) 7(6):e07236. 10.1016/j.heliyon.2021.e07236 34189295 PMC8219757

[22] Gasparro R Scandurra C Maldonato NM Dolce P Bochicchio V Valletta A Perceived Job Insecurity and Depressive Symptoms Among Italian Dentists: The Moderating Role of Fear of COVID-19. Int J Environ Res Public Health (2020) 17(15):E5338. 10.3390/ijerph17155338 32722202 PMC7432196

[23] Lam SC Arora T Grey I Suen LKP Huang EY Li D Perceived Risk and Protection from Infection and Depressive Symptoms Among Healthcare Workers in Mainland China and Hong Kong during COVID-19. Front Psychiatry (2020) 11:686. 10.3389/fpsyt.2020.00686 32765321 PMC7378321

[24] Al Ghafri T Al Ajmi F Anwar H Al Balushi L Al Balushi Z Al Fahdi F The Experiences and Perceptions of Health-Care Workers during the COVID-19 Pandemic in Muscat, Oman: A Qualitative Study. J Prim Care Community Health (2020) 11:2150132720967514. 10.1177/2150132720967514 33089729 PMC7585886

[25] Arnetz JE Goetz CM Arnetz BB Arble E. Nurse Reports of Stressful Situations during the COVID-19 Pandemic: Qualitative Analysis of Survey Responses. Int J Environ Res Public Health (2020) 17(21):E8126. 10.3390/ijerph17218126 33153198 PMC7663126

[26] Sridhar S Brouqui P Fontaine J Perivier I Ruscassier P Gautret P Risk Perceptions of MSF Healthcare Workers on the Recent Ebola Epidemic in West Africa. New Microbes New Infect (2016) 12:61–8. 10.1016/j.nmni.2016.04.010 27330816 PMC4900694

[27] Rush AJ Trivedi MH Ibrahim HM Carmody TJ Arnow B Klein DN The 16-Item Quick Inventory of Depressive Symptomatology (QIDS), Clinician Rating (QIDS-C), and Self-Report (QIDS-SR): a Psychometric Evaluation in Patients with Chronic Major Depression. Biol Psychiatry (2003) 54(5):573–83. 10.1016/s0006-3223(02)01866-8 12946886

[28] Spitzer RL Kroenke K Williams JB Lowe B. A Brief Measure for Assessing Generalized Anxiety Disorder: the GAD-7. Arch Intern Med (2006) 166(10):1092–7. 10.1001/archinte.166.10.1092 16717171

[29] Geoffrion S Lamothe J Morizot J Giguere CE. Construct Validity of the Professional Quality of Life (ProQoL) Scale in a Sample of Child Protection Workers. J Trauma Stress (2019) 32(4):566–76. 10.1002/jts.22410 31265178 PMC6771892

[30] Luan R Pu W Dai L Yang R Wang P. Comparison of Psychological Stress Levels and Associated Factors Among Healthcare Workers, Frontline Workers, and the General Public during the Novel Coronavirus Pandemic. Front Psychiatry (2020) 11:583971. 10.3389/fpsyt.2020.583971 33335490 PMC7736032

[31] Boyatzis R. Transforming Qualitative Information: Thematic Analysis and Code Development. Thousand Oaks, London & New Dehli: SAGE Publications (1998).

[32] Braun V Clarke V. Using Thematic Analysis in Psychology. Qual Res Psychol (2006) 3(2):77–101. 10.1191/1478088706qp063oa

[33] Malterud k. Qualitative Research: Standards, Challenges, and Guidelines. Lancet (2001) 358:483–8. 10.1016/S0140-6736(01)05627-6 11513933

[34] Cag Y Erdem H Gormez A Ankarali H Hargreaves S Ferreira-Coimbra J Anxiety Among Front-Line Health-Care Workers Supporting Patients with COVID-19: A Global Survey. Gen Hosp Psychiatry (2021) 68:90–6. 10.1016/j.genhosppsych.2020.12.010 33418193 PMC7749993

[35] Grailey K Lound A Brett S. Lived Experiences of Healthcare Workers on the Front Line during the COVID-19 Pandemic: a Qualitative Interview Study. BMJ Open (2021) 11(12):e053680. 10.1136/bmjopen-2021-053680 PMC8719006 35258477

[36] Sterling MR Tseng E Poon A Cho J Avgar AC Kern LM Experiences of Home Health Care Workers in New York City during the Coronavirus Disease 2019 Pandemic: A Qualitative Analysis. JAMA Intern Med (2020) 180(11):1453–9. 10.1001/jamainternmed.2020.3930 32749450 PMC7404061

[37] Olateju Z Olufunlayo T MacArthur C Leung C Taylor B. Community Health Workers Experiences and Perceptions of Working during the COVID-19 Pandemic in Lagos, Nigeria-A Qualitative Study. PLoS One (2022) 17(3):e0265092. 10.1371/journal.pone.0265092 35259204 PMC8903241

[38] Markkanen P Brouillette N Quinn M Galligan C Sama S Lindberg J It Changed Everything": The Safe Home Care Qualitative Study of the COVID-19 Pandemic's Impact on home Care Aides, Clients, and Managers. BMC Health Serv Res (2021) 21(1):1055. 10.1186/s12913-021-07076-x 34610836 PMC8491760

