# Supplementary material for: Risk Perception Related to COVID-19 and Future Affective Responses Among Healthcare Workers in Switzerland: A Mixed-Methods Longitudinal Study
Source: Int J Public Health. 2022 Sep 2;67:1604517. doi: 10.3389/ijph.2022.1604517 (PMC9478025; doi:10.3389/ijph.2022.1604517)
Supplement: Supplementary file 1 [file DataSheet1.pdf]

Appendix 1: Interview Grid (Risk perception related to covid-19 and future affective responses among healthcare workers in Switzerland: A mixed-methods longitudinal study; Switzerland. 2022)

- How would you describe your life during the pandemic?
    - Lived experience in general
    - Professional situation
    - Private situation

- Have you or anyone close to you been infected with COVID-19?
    - If so, how did it affect you or him/her?

- How did you experience the quarantine period (yours and/or a person close to you)?

- How was your work during the pandemic?
    - Changes (other assignments, teleworking, etc.)?
    - Description of a regular day?

- In general, what difficulties have you encountered during the pandemic (at work)?
    - What types of difficulties?
    - How did you feel during these difficulties?

- How did you manage these difficulties?
    - What resources did you use?
    - What help/support did you receive?
    - What did you find most helpful?
    - What would have helped more?

- In your professional setting, how often did you come into contact with patients infected OR suspected of being infected with COVID 19?
    - What type of contact have you had (direct, indirect, what management? context)?

- How did these contacts feel to you?
    - Can you recall any concrete cases?
    - What means of protection did you have?
    - What did you think of them?

- How did you feel when you were in contact with patients infected (OR suspected of being infected) with COVID-19?
    - Can you think of an actual case?
    - How did you feel in this situation (fear, stress, why)?

- During a health care interaction with a COVID-19 patient, have you had any accidents with body fluids/respiratory secretions?
    - Tell me how it happened? (feeling, experience etc.)

- What difficulties have you encountered when caring for patients infected with covid-19?
    - Examples
    - What helped you the most in this case?
    - What could have helped you more?
    - What resources did you mobilize?

- How did you generally feel at work during the pandemic?
  - What emotions did you have while coming to/from work?
  - How did you feel at the end of the day?
  - What habits did you change and why?

- What do you see as opportunities for improvement in handling a similar situation in the future?
  - What would you have needed to be better prepared?
  - What would have helped you more?
  - Other (e.g., training, specific type of intervention, other organization, etc.)?

- How would you describe the collaboration with your colleagues at the time of the pandemic?
  - How did this collaboration support you?
  - Difficulties encountered
  - Can you recall any concrete cases?
  - In your opinion, what are the possibilities for improvement?

- How was your collaboration with your superiors?
  - How did this collaboration support you?
  - Difficulties encountered?
  - Can you come back to concrete cases?
  - In your opinion, what are the areas for improvement?

- And how was your collaboration with the institution in general?
  - In what way did this collaboration bring you support?
  - Can you recall any concrete cases?
  - Difficulties encountered?
  - In your opinion, what are the areas of improvement?

- How is your work going now that the pandemic seems to have calmed down?
  - Are changes still present (e.g. reassignment)?

- You said you felt (this or that emotion), what about now?
  - What emotions do you feel when you come to work?
  - How do you feel at the end of a work day?

- How has this pandemic changed your perception of your profession and the way you practice it?
  - Are you satisfied or not with this change?
  - How has this been reflected in your attitude towards patients?
  - What changes do you notice in your collaboration with your colleagues?

- What other elements would you like to share?
